# Postural effects of dental loupes in pediatric dental practice?

**DOI:** 10.1186/s12903-025-05766-0

**Published:** 2025-03-26

**Authors:** Rumeysa Çınar, Sema Aydınoğlu, İpek Arslan, Dilara Nil Günaçar

**Affiliations:** 1 Department of Pediatric Dentistry, Faculty of Dentistry Recep, Tayyip Erdoğan University, Rize, Turkey; 2 Assoc. Prof, Specialist Pediatric Dentist, Sakarya, Turkey; 3 Department of Oral and Maxillofacial Radiology, Faculty of Dentistry, Recep Tayyip Erdoğan University, Rize, 53100 Turkey

**Keywords:** Dentists, Ergonomics, Musculoskeletal diseases, Pediatric dentistry, Posture

## Abstract

**Background:**

Pediatric dentists may encounter musculoskeletal disorders (MSD) when they cannot maintain neutral postures during dental treatments. It is known that dental loupes (DL) can contribute to improving the ergonomic posture of dentists during treatment. This study aims to investigate the effect of wearing DL on the working posture of pediatric dentists.

**Methods:**

In pulpotomy procedures performed with DL in 23 patients and without DL in 23 patients, photographs were taken at a total of 3 times in each patient: at the beginning of the procedure, 15^th,^ and 30^th^ minutes. The working postures of the clinicians were analyzed using the Modified-Dental Operator Posture Assessment Instrument (M-DOPAI) method through these photographs. The differences between the working postures of pediatric dentists in both groups were analyzed using the Wilcoxon test. The Friedman test determined the statistical relationship between pediatric dentists’ time-dependent working postures.

**Results:**

At the beginning, 15^th^, and 30^th^ minutes of treatment, the neck and shoulder postures and the total M-DOPAI scores of clinicians using DL were significantly lower than those working without DL (*P* < 0.05). However, no significant difference was found among the total scores determined at different time intervals during the treatment (*P* > 0.05).

**Conclusions:**

The use of dental loupes has been proven to lower ergonomic risk levels related to the working postures of pediatric dentists. By promoting a more neutral neck and shoulder position, dental loupes may help reduce the risk of occupational musculoskeletal disorders.

**Trial registration:**

The trial protocol was retrospectively registered ID NCT06799585 (https://clinicaltrials.gov/); Jan 29, 2025.

## Background

Musculoskeletal disorders (MSD) are injuries and diseases affecting muscles, nerves, tendons, ligaments, joints, cartilage, spinal discs, and blood vessels. Problems related to MSD can occur in the back, hips, wrists, shoulders, head, or neck area [1–4]. Dentists work with limited visual sight, so their movement is limited, and prolonged static poor postures may be repeated. During this time, MSD may develop as an occupational disease due to repetitive, straining movements, overhead work, overuse of arms and hands, excessive use of the body in incorrect posture, and ergonomic deficiencies [5–9]. While mild forms of MSD may be temporary, asymptomatic, or even undiagnosed, severe forms of MSD can cause serious adverse effects, including irreversible and persistent pain, functional limitations, and disability that affect daily activities, quality of life, ability to earn a living and independence [8]. When working by leaning forward, the cervical spine is in forward flexion and rotation, which puts pressure on the shoulder joint and shoulder-neck muscles. This situation creates serious strain on the bone structure and soft tissues of the neck and shoulder area; it leads to MSD problems such as thoracic outlet syndrome, rotator cuff tendonitis, and myofascial pain. Dentists who have to work in this way may experience MSD more frequently as a result of working positions that do not comply with ergonomic rules [10, 11]. Studies have shown that MSD is detected in dentists at rates ranging from 4.8% [9, 12] to 82% [9, 13]. Considering the high rates, it can be seen that maintaining a neutral body posture has an extremely important effect on the clinician’s general health [14, 15]. Magnification systems and lighting devices, which affect the physician’s field of vision and working position, play an important role in ensuring an ergonomic posture. Magnification tools such as the dental operating microscope (DOM) and the dental loupe (DL) are useful in neutralizing practitioners’ posture, as well as allowing them to perform precise work in science, and especially dentistry [16–18].

There are many methods in the literature to determine the abnormal working postures that cause MSD and the risk levels that these situations create in the body [19, 20]. Modified-Dental Operator Posture Assessment Instrument (M-DOPAI), which was created by combining the Posture Assessment Instrument (PAI) tested for validity and reliability by Branson et al. [21] and the Posture Assessment Criteria (PAC) prepared by Maillet et al. [16], is a method used to evaluate working posture positions created by Partido [22].

There are studies in the literature evaluating the posture of dentistry students using DL and different chairs on phantom models [23], studies evaluating the changes in the posture of dental hygienists using DL, or studies evaluating the posture of dentists and chairside dental assistants during the procedure [24]. This study, which will be the first in the literature, aimed to evaluate the postures of pediatric dentists using dental loupes at different times of intraoperative interventions with the M-DOPAI method. The null hypothesis was that there would be no difference in observational ergonomic scores between pediatric dentists using dental loupes and those not using dental loupes when their postures were evaluated using M-DOPAI.

## Materials and methods

### Ethical statement

Recep Tayyip Erdoğan University Faculty of Medicine Non-Interventional Clinical Research Ethics Committee (2023/251) approved this study. Before the study, participants were given detailed information about the research, and an informed consent form was obtained. According to the principles described in the Declaration of Helsinki, the study protocol included all amendments and revisions.

### Study group selection

Power analysis was conducted using the G*Power 3.1.0 software package (Universität Düsseldorf, Germany) to determine the required sample size for the study. According to the power analysis performed to determine the number of volunteers to be included in the study, it was determined that the research should be conducted on at least 20 participants with 95% confidence (1-α), 95% test power (1-β), and w = 0.769 effect size [25]. However, considering possible missing data, it was decided to include 23 participants in the study. The clinicians included in the study were female pediatric dentists who were research assistants at the Department of Pediatric Dentistry, Faculty of Dentistry, Recep Tayyip Erdoğan University, had < 5 years of endodontic work experience, performed pedodontic treatments more than 4 days a week, had no experience in using dental loupes, were between the ages of 25–30, were right-handed, did not have any MSD, and volunteered to participate in the study. Clinicians with a history of trauma, accident, or surgery in the neck region, MSD or psychosomatic disorder in the last 6 months, body mass index (BMI) greater than 25 kg/m^2^, left-handed, or pregnant were excluded from the study. The patients included in the study consisted of pediatric patients aged between 6 and 8 years who had an indication for pulpotomy in the mandibular right second primary molar, were 3 (positive)- 4 (definitely positive) according to the Frankl Behavior Scale [26], whose parents volunteered to participate in the study and signed the informed consent form, and who did not have any systemic disease or disability. A digital software tool, Randomizer (available at www.randomizer.org), was utilized to randomly assign participants to the study groups (accessed on January 15, 2024).

### Procedure

**Fig. 1:**
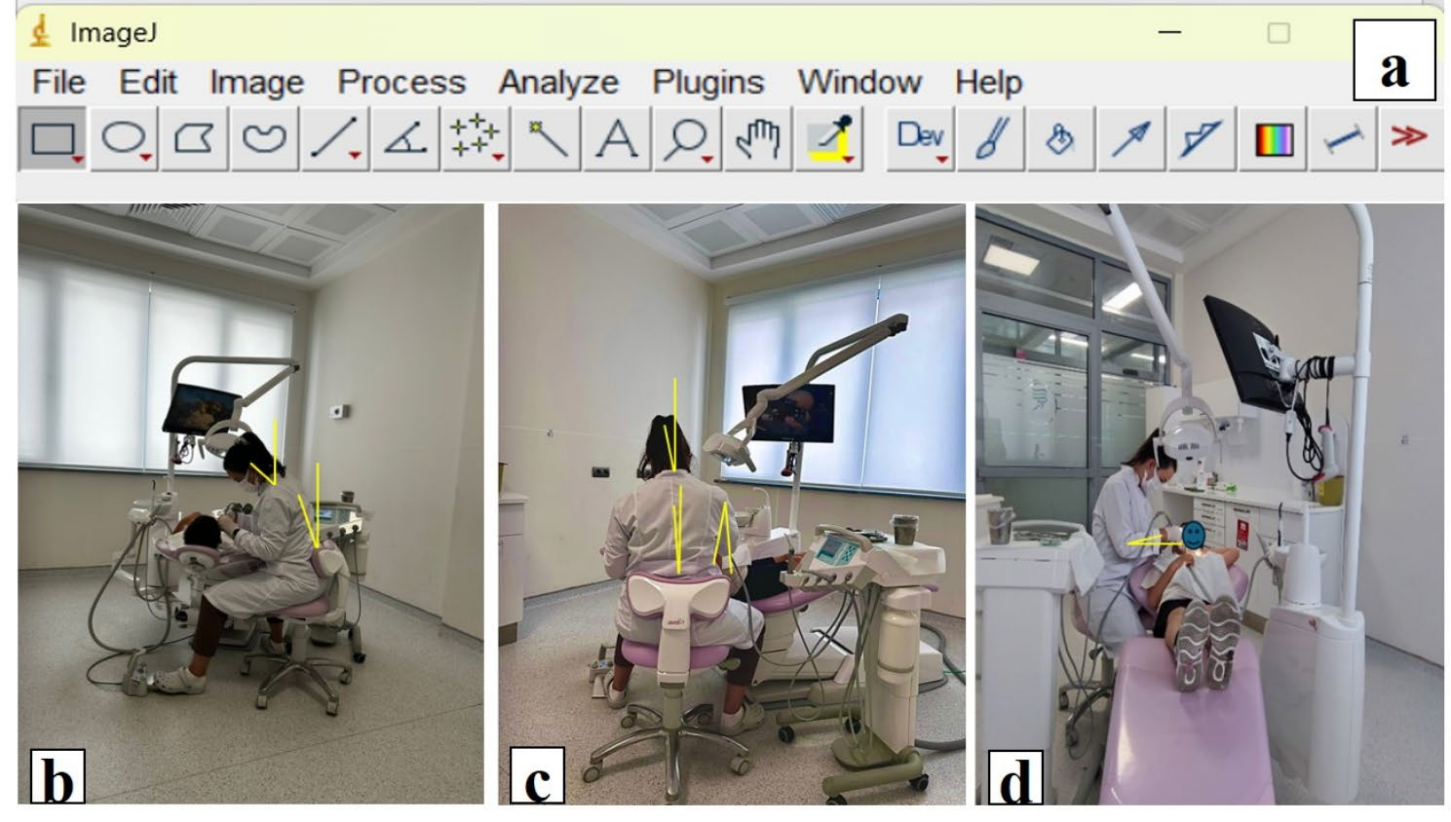
Representation of measurements on photographs for Modified-Dental Operator Posture Assessment Instrument **(a)** ImageJ program **(b)** Pediatric dentist’s neck (front to back) angle, trunk (front to back) angle **(c)** Pediatric dentist’s neck (side to side) angle, trunk (side to side) angle, and upper arm (parallel) **(d)** Pediatric dentist’s upper arm (elbow) angle

Treatment appointments were set to be at the first hour of the day to ensure standardization for each patient. The physicians worked with the same patient chair, physician chair, and DL. During the photo shoot, a green color line laser source (Bosch Universal Level 360, Germany) with a working area diameter of 24 m was used to determine the planes. The photos were taken with a professional phone camera without flash or sound (Xiaomi 11T Pro camera, Xiaomi, China). Clinicians in the study performed pulpotomy treatments of 23 patients using the DL x2.5 magnification (Dental Loupes, NSKI, China) (PwDL) and pulpotomy treatments of the other 23 patients without using the DL (PwoDL). Accordingly, photographs of the pediatric dentists were taken at the beginning of the treatment, 15th and 30th minutes while they were at the patient’s chairside. The photographs were taken from three different places where the clinicians’ working postures could be seen. Accordingly, each clinician was photographed three times in total; approximately 1.5 m from her left side, in line with her body and perpendicular to the ground; approximately 1.5 m from her right side, in line with her body and perpendicular to the ground; and approximately 1.5 m from her back, in line with her body and perpendicular to the ground. Then, the working postures of the clinicians were evaluated on the photographs taken using M-DOPAI method. ImageJ software (https://imagej.nih.gov/ij/ accessed on 4 January 2024 developed by Wayne Rasband, National Institutes of Health, Bethesda, MD, USA) was used to measure the clinicians’ working position angles from the photographs (Fig. 1). Before assessing the MDOPAI evaluation from the photographs, each image was numbered, and the area around the eyes was blurred to conceal whether the dentists were using dental loupes. In this way, the evaluator was blinded and no bias occurred.

### Modified-dental operator posture assessment instrument (M-DOPAI)

M-DOPAI is a 12-component assessment method that can evaluate the postures of the hips, trunk, head/neck, upper arms, shoulders, and wrists during work [22]. This method is a specific and combined version of the 10 components of Branson et al.‘s PAI [21] and 2 components of Maillet et al.‘s PAC [16] for evaluating dental operator posture. A score of 12 defines the ideal posture. In this assessment, which is scored between 12 and 32, the further the operator is from the ideal posture, the higher the total score [22]. Since posture assessment was made through photographs in this study method and therefore the rotation movement between the planes could not be determined through photographs, head-neck and trunk rotation assessments that should be measured in M-DOPAI could not be made. For the postures of the upper arm and wrist, only the right extremities of the clinicians were analyzed.

### Data analysis

Data were recorded into a spreadsheet program before being analyzed by SPSS. The data were analyzed using SPSS v23.0 (SPSS Inc, Chicago, IL, ABD). The normal distribution of the continuous data was evaluated with the Shapiro–Wilk test. Since the data of the study groups did not show a normal distribution, Wilcoxon tests (DL vs. without DL) were used to assess differences in each dependent variable. For time-dependent analyses within the groups (beginning, 15 ^th^, and 30^th^ minutes), the Friedman test was applied because the data did not conform to a normal distribution. Significance levels were set at values < 0.05.

For the evaluation of intraobserver agreement, Cohen’s κ was used for this purpose, the clinicians’ posture scores were re-scored after one week.

## Results

A total of 414 photographs were taken of 23 pediatric dentists during both pulpotomy procedures with and without a dental loupe. Images were captured at three-time points (beginning, 15^th^ minute, and 30^th^ minute), with three different exposures taken at each time: right side, left side, and back side. The age range of the 23 female pediatric dentists participating in the study was 25–30 years, and the mean age was 27.87 ± 1.66 years. The weighted κ coefficients were found to be 0.817, with strong intraobserver reliability [27].

The average M-DOPAI score values ​​of the clinicians’ working postures at the beginning, 15^th^, and 30^th^ minutes of the treatment are given in Table 1. Accordingly, in the treatments performed using DL, the clinicians’ neck forward and lateral flexion, and shoulder postures received significantly lower scores compared to the clinicians who did not use DL (*P* < 0.001; *P* = 0.001; *P* < 0.001; *P* = 0.033). However, in the treatments performed using DL, the clinicians’ wrist posture scores were found to be significantly higher compared to the clinicians who did not use DL (*P* = 0.008). The total M-DOPAI score obtained from the working postures of the clinicians who did not use DL (16.47 ± 1.59) was significantly higher compared to the clinicians who used DL (14.52 ± 0.89) (*P* < 0.001).

**Table 1:** According to the M-DOPAI method, the score values ​​of the pediatric dentists’ working postures at the beginning, 15^th^, and 30^th^ minutes of the treatment

	Time		PwoDL Mean ± SD M (Min-Max)	PwDL Mean ± SD M (Min-Max)	*P*
Hips (leveled)	0	1.04 ± 0.20 1 (1–2)		1.13 ± 0.34 1 (1–2)	0.157
	15	1.13 ± 0.34 1 (1–2)		1.26 ± 0.44 1 (1–2)	0.257
	30	1.13 ± 0.34 1 (1–2)		1.13 ± 0.34 1 (1–2)	1.000
Trunk (front to back)	0	1.08 ± 0.28 1 (1–2)		1.00 ± 0 1 (1–1)	0.157
	15	1.08 ± 0.28 1 (1–2)		1.00 ± 0 1 (1–1)	0.157
	30	1.13 ± 0.34 1 (1–2)		1.00 ± 0 1 (1–1)	0.83
Trunk (side to side)	0	1.00 ± 0 1 (1–1)		1.00 ± 0 1 (1–1)	1.000
	15	1.00 ± 0 1 (1–1)		1.00 ± 0 1 (1–1)	1.000
	30	1.00 ± 0 1 (1–1)		1.00 ± 0 1 (1–1)	1.000
Neck (front to back)	0	2.91 ± 0.28 3 (2–3)		2.04 ± 0.20 2 (2–3)	< 0.001*
	15	2.91 ± 0.28 3 (2–3)		2.00 ± 0 2 (2–2)	< 0.001*
	30	2.95 ± 0.20 3 (2–3)		2.17 ± 0.38 2 (2–3)	< 0.001*
Neck (side to side)	0	1.56 ± 0.58 2 (1–3)		1.04 ± 0.20 1 (1–2)	0.001*
	15	1.43 ± 0.58 1 (1–3)		1.00 ± 0 1 (1–1)	0.004*
	30	1.26 ± 0.44 1 (1–2)		1.00 ± 0 1 (1–1)	0.014*
Upper arm (parallel)	0	2.34 ± 0.57 2 (1–3)		2.30 ± 0.55 2 (1–3)	0.705
	15	2.39 ± 0.65 2 (1–3)		2.21 ± 0.51 2 (1–3)	0.248
	30	2.26 ± 0.61 2 (1–3)		2.26 ± 0.54 2 (1–3)	1.000
Upper arm (elbow)	0	1.82 ± 0.38 2 (1–2)		1.91 ± 0.28 2 (1–2)	0.414
	15	1.82 ± 0.38 2 (1–2)		1.95 ± 0.20 2 (1–2)	0.083
	30	1.86 ± 0.34 2 (1–2)		1.91 ± 0.28 2 (1–2)	0.564
Shoulders (slumped/relaxed)	0	1.60 ± 0.49 2 (1–2)		1.00 ± 0 1 (1–1)	< 0.001*
	15	1.78 ± 0.42 2 (1–2)		1.00 ± 0 1 (1–1)	< 0.001*
	30	1.65 ± 0.48 2 (1–2)		1.00 ± 0 1 (1–1)	< 0.001*
Shoulders (leveled)	0	1.52 ± 0.51 2 (1–2)		1.17 ± 0.38 1 (1–2)	0.033*
	15	1.82 ± 0.38 2 (1–2)		1.26 ± 0.44 1 (1–2)	0.001*
	30	1.82 ± 0.38 2 (1–2)		1.34 ± 0.48 1 (1–2)	0.005*
Wrist (flexed/extended)	0	1.60 ± 0.49 2 (1–2)		1.91 ± 0.28 2 (1–2)	0.008*
	15	1.65 ± 0.48 2 (1–2)		1.78 ± 0.42 2 (1–2)	0.366
	30	1.69 ± 0.47 2 (1–2)		1.69 ± 0.47 2 (1–2)	1.000
Total Score	0	16.47 ± 1.59 16 (14–19)		14.52 ± 0.89 14 (13–17)	< 0.001*
	15	17.04 ± 1.06 17 (15–19)		14.47 ± 0.73 15 (13–16)	< 0.001*
	30	16.78 ± 1.24 17(15–20)		14.52 ± 0.89 14 (13–17)	< 0.001*

*: There was a significant difference between groups with the Wilcoxon test (significance set at *P*-values < 0.05); SD: Standard deviation; M: Median; Min: Minimum, Max: Maximum; PwoDL: Pulpotomy without dental loupe; PwDL: Pulpotomy with dental loupe

At the 15^th^ minute of treatment, the mean scores of forward and lateral flexion of the neck, and shoulder postures were found to be significantly lower in clinicians using DL (*P* < 0.001; *P* = 0.004; *P* < 0.001; *P* = 0.001). While the total M-DOPAI score obtained from the working postures of clinicians who did not use DL was 17.04 ± 1.06, the total M-DOPAI score in those using DL was found to be 14.47 ± 0.73. Accordingly, the total M-DOPAI scores of clinicians who performed treatment using DL were significantly lower (*P* < 0.001).

At the 30^th^ minute of the treatment, the mean score values ​​determined in the forward and lateral flexion of the neck, and shoulder postures were lower in the DL group (*P* < 0.001; *P* = 0.014; *P* < 0.001; *P* = 0.005). While the total M-DOPAI score obtained from the working postures of the clinicians who did not use DL was 16.78 ± 1.24, the total M-DOPAI score determined in the treatments performed using DL was 14.52 ± 0.89. The total score values ​​calculated for the working postures of the clinicians who worked using DL were found to be significantly lower compared to the clinicians who worked without DL (*P* < 0.001).

Total M-DOPAI score values ​​of the postures of the dentists who used and did not use DL at the beginning, 15^th^, and 30^th^ minutes of the treatment are given in Table 2. Accordingly, it was found that the total M-DOPAI scores of the postures of both the dentists who did not use DL and those who used DL did not show any change at the beginning, 15^th^, and 30^th^ minutes of the treatment (*P* > 0.05).

**Table 2:** Total point values ​​of pediatric dentists’ postures at the beginning, 15^th^, and 30^th^ minutes of treatment, according to the M-DOPAI method

	PwoDL Mean ± SD M (Min-Max)	PwDL Mean ± SD M (Min-Max)
Beginning	16.47 ± 1.59 16 (14–19)	14.52 ± 0.89 14 (13–17)
15^th^	17.04 ± 1.06 17 (15–19)	14.47 ± 0.73 15 (13–16)
30^th^	16.78 ± 1.24 17 (15–20)	14.52 ± 0.89 14 (13–17)
*P*	0.172	0.869

The difference between the groups was examined with the Friedman test and significance set at *P*-values < 0.05; SD: Standard deviation; M: Median; Min: Minimum, Max: Maximum; PwoDL: Pulpotomy without dental loupe; PwDL: Pulpotomy with dental loupe

## Discussion

The null hypothesis was rejected. It was observed that the total M-DOPAI scores, measured at three different time points during pulpotomy treatments, significantly decreased with the use of dental loupes. Additionally, neck and shoulder ergonomic scores were notably closer to a neutral posture.

MSD, which may require medical support, reduce work efficiency, and negatively impact the quality of life, can occur especially in dentists due to their limited range of motion and abnormal working postures in a narrow area [8]. Studies are reporting that MSD in dentists begin during undergraduate education [28–30]. In a study conducted by Altaş et al., the working postures of dentistry students doing internships in different branches were evaluated and it was reported that dentistry students doing pedodontics internships showed the most ergonomically risky postures [31]. Jahanimoghadam et al. evaluated the ergonomic risk analysis of 90 specialist dentists in their study and reported that 90% of the participants had a medium/high level of risk regarding their working positions. They also reported that the physicians with the highest risk group were pediatric dentists, periodontists, and oral maxillofacial surgeons [32]. The reasons why pediatric dentists work in poor ergonomic postures during treatment include the limited field of vision of the child’s mouth, problems in cooperating with children, and difficulties in positioning the patient in the correct position. Although such problems are frequently encountered, it is known that studies examining the working postures of pedodontists from an ergonomic perspective are limited. There are studies in the literature reporting that dentists who work using appropriate DL have reduced neck and back pain [33–36]. There are also studies supporting this that the use of DL reduces the degree of forward flexion and increases visual acuity when working [16, 18, 33, 37]. In addition, James and Gilmour stated in their study that dentists can work in a more upright posture with the selection, adjustment, and use of a correct magnification system [37]. Therefore, the effect of wearing DL on the working postures of pedodontic residents was evaluated in this study.

In the literature, many observational methods have been used to determine the abnormal working postures of dental hygiene students that cause MSD and the risk levels that these situations pose to the body [16, 18, 22]. Observational methods are more widely preferred due to their low cost, acceptable sensitivity levels, and reliability [38]. The PAI method, whose validity and reliability were performed by Branson et al. in 2002, is used for the ergonomic evaluation of the displayed and real-time postures of dentists and dental hygienists [21]. The PAC method, developed by Maillet et al., was created by modifying the PAI method [16]. Another method used in dentistry, M-DOPAI, developed by Partido, was obtained by combining PAI and PAC. In a study conducted by Partido and Wright, the working postures of dentistry students were evaluated using the M-DOPAI method, and the students were also allowed to make ergonomic self-assessments based on their working postures [39]. This study has shown that posture awareness among dentistry students has increased and that improvement has been achieved in line with students’ self-assessments [39]. M-DOPAI was chosen for this study in ergonomic risk assessment because it is a method developed specifically for dentists, emerged as a result of the development of PAI and PAC methods, and is a low-cost, acceptable and reliable method. According to this method, in this study, photographs were taken of the participants throughout the treatment, and posture assessment was performed using the M-DOPAI method on the obtained photographs.

There are studies in the literature where dentists’ ergonomic risk levels are evaluated with many different methods using magnification systems. A study in which an endodontist’s working posture was examined with the Rapid upper limb assessment (RULA) method, in which he opened an endodontic access cavity for tooth number 47 of three different patients using an operating microscope and DL, and without using any magnification system, showed that the RULA scores of the dentist were similar in procedures performed with and without a DL; however, the average score in the procedure performed with the operating microscope was lower [40]. In the study conducted by Branson et al. on dental hygienist students, the experimental group performed the probing procedure using DL, while the other group performed the same procedure with protective glasses. In this study in which the participants’ working positions were evaluated using the PAI method, the total score values ​​of the working postures of the students using DL were found to be significantly lower than those not using DL [18]. In another study, the working postures of dental hygienist students who performed scaling/root planing procedures both with and without DL were evaluated with the PAC method, and according to this, the group performing periodontal procedures using DL obtained significantly lower ergonomic scores compared to the group working without using DL. As a result of the study, it was reported that the use of DL reduced ergonomic scores, allowing the participants to approach the ideal working posture and improve their working postures [16]. In addition, Kamal et al. stated that the use of DL positively affected the working postures of the students in a preclinical environment in their study where they examined the working postures of dentistry students [41]. In a study conducted by Dable et al., the working postures of dentistry students were evaluated with the RULA method, and as a result, it was observed that the students using DL worked in postures that were ergonomically lower risk than those not using DL [23]. Studies have shown that the use of DL contributes positively to the working postures of physicians who have different experiences and perform different dental treatments. This study also concluded in parallel with previous studies in the literature and found that the use of DL positively affects the working postures of physicians. It is thought that this situation is because dentists using DL can work without having to bend over more, as they provide a good view.

In the study by Hayes et al., dental hygienists using DL were found to have lower shoulder and wrist pain than those not using them [42]. In the current study, DL may be thought to reduce the likelihood of dental hygienists experiencing shoulder pain, as DL had lower shoulder posture scores. In a study by Hugues and Adorna, arm and wrist RULA scores measured when a dentist worked with or without a DL were similar [40]. However, in the current study, it was observed that the score values ​​determined according to the wrist of dentists using DL at the beginning of the treatment were higher. In the 15^th^ and 30^th^ minutes of the treatment, no significant difference was found between the scores determined according to the wrist postures of the dentists using DL. It is believed that this could be due to the dentists in the study not being able to adapt to the dental loupes at the start of the treatment, as they had no experience with them.

In the study conducted by Carpentier et al., the working postures of dentistry students while preparing cavities on phantom models with and without DL were examined [43]. The postures of the students were evaluated using the modified PAI method; trunk, head-neck, and upper arm postures were analyzed with this method. Accordingly, students who used DL received scores indicating lower ergonomic risk levels compared to those who did not use. This situation, similar to the current study, shows that the use of DL allows dentists to work in a position close to a neutral posture. In line with the current study, no significant relationship was observed in the scores calculated for the upper arm region of the participants based on the use of DL. In the same study, it was reported that the scores calculated for trunk posture were significantly higher when DL were not used [43]. In the current study, no significant difference was detected in the trunk postures of the dentists depending on their use of DL. It is thought that the difference between the findings of the two studies is because the current study was conducted in a clinical setting, the population consisted of pediatric patients, and the treatment procedure was a complex treatment that took longer than cavity preparation.

Adulyawat et al. examined the neck forward flexion postures of dentists with less than 5 years of experience in the field of endodontics during root canal treatment without wearing DL using the M-DOPAI method [44]. As a result of this study, it was stated that the average scores of the dentists regarding neck forward flexion postures in the last stage of root canal treatment were higher than in the previous stages. These findings revealed that the endodontists who participated in the study worked by bending their necks more forward toward the end of the treatment. In the current study, when the working postures of the pedodontics residents were examined at different times of the treatment, no significant difference was found between the determined total scores. It is thought that this situation may be due to the longer treatment duration of the endodontists compared to this study and their working on the upper jaw. It is also thought that the posture evaluations made at different times of the treatment were made based on the total score in the current study and only using neck forward flexion values ​​in the other study may have an effect.

The limitations of the study include the evaluation of participants’ working postures only through photographs using observational measurement methods. This may cause individuals to work by correcting their working postures and may prevent real results from being obtained. However, it is seen in the literature that most of the ergonomic analyses conducted in the field of dentistry are performed in preclinical settings and on phantom jaws. Also, dental loupe usage experience can impact clinicians’ posture. This study examined posture changes in physicians with no prior experience using dental loupes. Future research can explore posture differences based on varying durations of loupe usage. One of the strongest aspects of the current study is that the postures of pedodontic residents who apply a standardized treatment protocol in a clinical setting are evaluated at different time intervals. In addition, there is no study in the literature evaluating the effect of DL on the working postures of pedodontics residents at different time intervals. With these aspects, it is thought that the current study will make significant contributions to the literature. In addition, new studies can be planned by expanding the sample size and using different ergonomic analysis methods.

## Conclusion

When properly adjusted for working distance and inclination angle, dental loupes help pediatric dentists maintain a more neutral neck and shoulder posture. Their use has been shown to reduce ergonomic risk levels associated with dentists’ working postures, potentially decreasing the risk of occupational musculoskeletal disorders.

## Data Availability

The datasets used and/or analysed during the current study are available from the corresponding author on reasonable request.

## Abbreviations

BMI: Body Mass Index
DL: Dental Loupe
DOM: Dental Operating Microscope
M-DOPAI: Modified-Dental Operator Posture Assessment Instrument
MSD: Musculoskeletal Disorders
PAC: Posture Assessment Criteria
PAI: Posture Assessment Instrument
PwDL: Pulpotomy with Dental Loupe
PwoDL: Pulpotomy without Dental Loupe
RULA: Rapid Upper Limb Assessment
SPSS: Statistical Package for the Social Sciences
κ: Cohen’s Kappa (used for intraobserver agreement)
